# Regulation of myocardial contraction as revealed by intracellular Ca^2+^ measurements using aequorin

**DOI:** 10.1186/s12576-024-00906-7

**Published:** 2024-02-21

**Authors:** Satoshi Kurihara, Norio Fukuda

**Affiliations:** Department of Cell Physiology, The Jikei University School of Medicine, 3-25-8 Nishi-Shimbashi, Minato-Ku, Tokyo, 105-8461 Japan

**Keywords:** Calcium, Excitation–contraction coupling, Heart, Muscle

## Abstract

Of the ions involved in myocardial function, Ca^2+^ is the most important. Ca^2+^ is crucial to the process that allows myocardium to repeatedly contract and relax in a well-organized fashion; it is the process called excitation–contraction coupling. In order, therefore, for accurate comprehension of the physiology of the heart, it is fundamentally important to understand the detailed mechanism by which the intracellular Ca^2+^ concentration is regulated to elicit excitation–contraction coupling. Aequorin was discovered by Shimomura, Johnson and Saiga in 1962. By taking advantage of the fact that aequorin emits blue light when it binds to Ca^2+^ within the physiologically relevant concentration range, in the 1970s and 1980s, physiologists microinjected it into myocardial preparations. By doing so, they proved that Ca^2+^ transients occur upon membrane depolarization, and tension development (i.e., actomyosin interaction) subsequently follows, dramatically advancing the research on cardiac excitation–contraction coupling.

## Historical background

Sydney Ringer, a British physiologist and clinician, was born in 1835 in England, and studied medicine at University College London, from 1854 and graduated in 1860. His most lasting contribution to physiology was conducted at the Department of Physiology of University College London; viz*.*, he demonstrated that the existence of the chloride salt of Ca^2+^ is essential for sustained spontaneous beating of the isolated frog heart [59]. His demonstration of the necessity for extracellular Ca^2+^ for the beating of the frog heart was the first to directly indicate the physiological importance of Ca^2+^ in cardiac muscle contraction. This important work provided the basis of the well-known Ringer’s solution (developed in 1882; e.g., [54]), i.e., an aqueous solution containing chloride salts of Na^+^, K^+^ and Ca^2+^ that provides a medium which is essentially isotonic to many animal tissues. After Ringer’s work, Langendorff in 1895 insisted on the use of oxygenated blood to effectively perfuse the isolated mammalian hearts [49], and Gremels and Starling demonstrated in 1926 that an adequate level of oxygen must be dissolved in the perfusate of mammalian hearts [26].

It was Pollack in 1928 who first realized that in order to clarify the regulatory role of intracellular Ca^2+^, its concentration must be measured in living cells [58]. Indeed, Pollack injected the dye alizarin sulphonate into an amoeba, observed brick-red deposits (see [50] for chemical property of alizarin sulphonate which reacts with Ca^2+^ to form salt) adjacent to the sites of pseudopod formation in the amoeba, and concluded that the intracellular Ca^2+^ concentration ([Ca^2+^]_i_) was increased in these sites. The specificity of this early method for Ca^2+^ may have been criticized; however, Pollack’s work evoked other scientists to consider the role of intracellular Ca^2+^ in various cellular regulations.

In 1940, Heilbrunn showed that when isolated frog muscle fibers are immersed in the solution containing CaCl_2_, they rapidly and markedly shorten [29]. Then, Kamada and Kinoshita [34] and Heilbrunn and Wiercinski [30] independently showed that localized contraction, induced in a fresh single muscle fiber, slowly longitudinally spreads when its sarcolemma is injured by pinching with a forceps or tearing with an injection pipette. It is important to note that this phenomenon was observed only when the solution contained Ca^2+^. These observations suggest that Ca^2+^ plays a pivotal role in the contraction of muscle fibers.

By the late 1950s, evidence had been mounting, by using various muscle model systems, that the hydrolysis of ATP is essential in both contraction and relaxation of muscle. In 1959, Weber showed, in a well-organized study using the Ca^2+^ chelator EGTA, that an increase in the Ca^2+^ concentration from ~ 1 to ~ 10 µM induces the activation of myofibrillar MgATPase [73]. Soon after that work by Weber, Ebashi and Lipmann [19] discovered that the relaxing factor, derived from the sarcoplasmic reticulum (SR), accumulates Ca^2+^ in the presence of ATP. It is interesting that in those days it was rather an unpopular theory especially among biochemists who refused to accept that Ca^2+^, a simple inorganic ion, controls a physiologically important phenomenon of muscle contraction and relaxation. However, Ebashi and Kodama made the groundbreaking discovery of troponin (Tn), the first identified Ca^2+^ receptor, which mediated Ca^2+^ control of muscle contraction [17, 18]. Moreover, they showed that the relaxing protein called “native tropomyosin” [15], isolated from muscle extracts, is a copolymer or a complex of tropomyosin (Tm) and Tn.

**Fig. 1:**
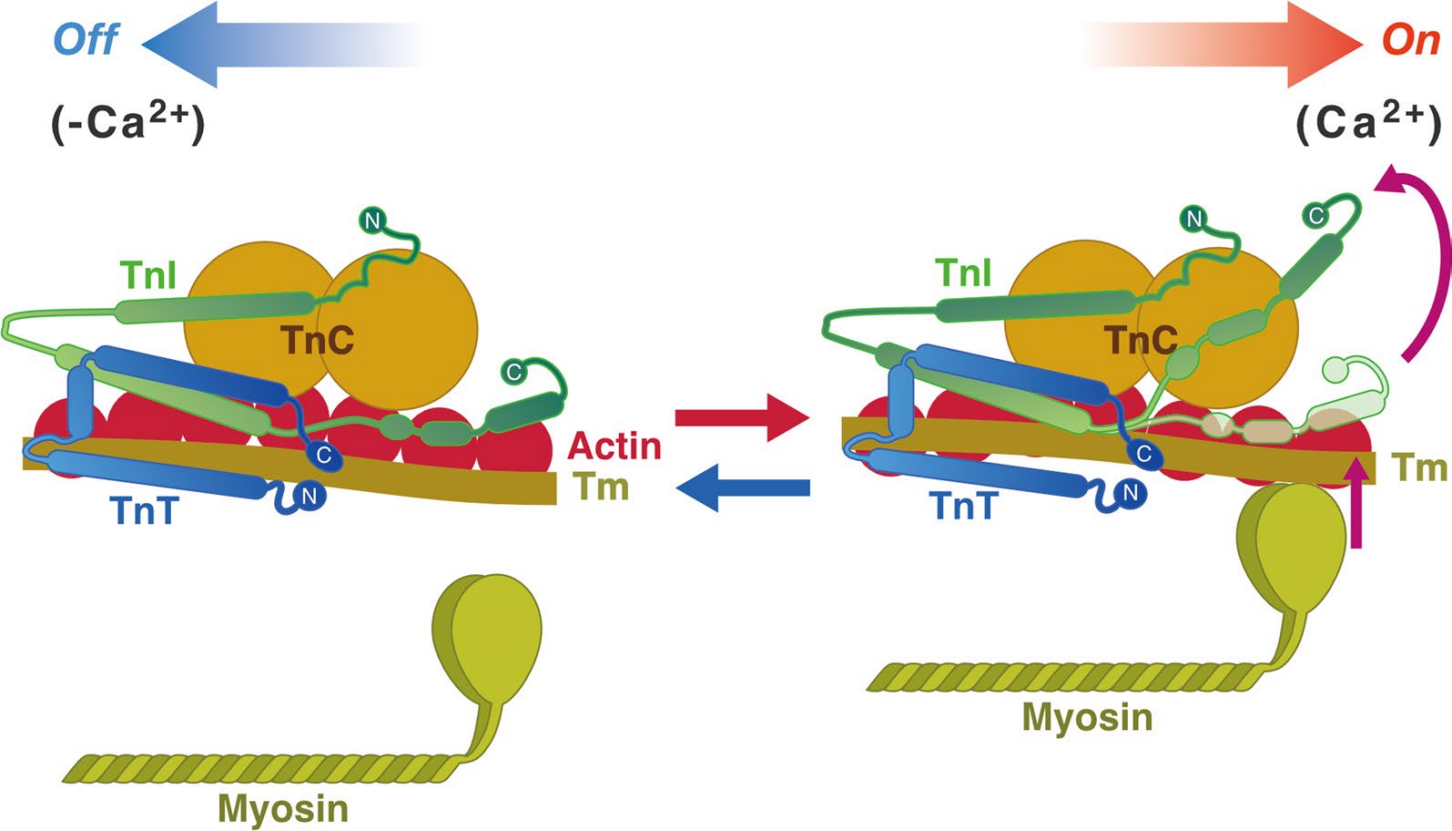
Structure and arrangement of cardiac thin filament proteins in the absence and presence of Ca^2+^. Upon Ca^2+^ binding to TnC, the C-terminus region of TnI dissociates from actin, allowing for Tm movement and, consequently, myosin binding to actin (indicated by arrows). Tm, tropomyosin; TnT, troponin T; TnI, troponin I; TnC, troponin C. C, COOH terminus; N, NH_2_ terminus. The equilibrium between the “*off*” state and the “*on*” state is a function of [Ca^2+^]_i_. H^+^ competes with Ca^2+^ for the regulatory Ca^2+^-binding site of TnC and thereby apparently inhibits the actomyosin interaction. Modified from Fig. 3 of [38] in an open access article published by Springer Nature with permission.

Currently, it is well established, in both cardiac and skeletal muscles, that the state of myofilaments is regulated by the Tm-Tn complex on thin filaments, depending on [Ca^2+^]_i_ (e.g., [16, 24, 38, 55, 66]) (see Fig. 1 for the structure and arrangement of cardiac thin filament proteins). Tn is a heterotrimer of TnC, TnI, and TnT [20, 28, 60]. Two metal binding sites are present in the C-terminal domain of TnC that bind both Mg^2+^ and Ca^2+^ with a relatively high affinity. Because Mg^2+^ exists at relatively high concentrations inside cardiomyocytes (~ 1 mM compared with ~ 0.1 to ~ 1 µM for Ca^2+^), these sites are occupied by Mg^2+^ under physiological conditions. While fast skeletal TnC has two regulatory Ca^2+^-binding sites in the N-terminal domain of TnC, cardiac TnC has only one regulatory Ca^2+^-binding site. When [Ca^2+^]_i_ increases during activation, Ca^2+^ binds to the regulatory Ca^2+^-binding site, resulting in the onset of the conformational change of the thin filament. At the resting state, the C-terminal domain of TnI tightly binds to actin, and Tm blocks the actomyosin interaction (“*off*” state). However, when Ca^2+^ binds to the regulatory Ca^2+^-binding site of TnC during activation, the C-terminal domain of TnI is dissociated from actin, and binds to the N-terminal domain of TnC, due to the enhanced TnC-TnI interaction (“*on*” state). The transition from the “*off*” to “*on*” state is considered to be associated with the movement of Tm on the thin filament [52, 67]. It should also be noted that H^+^ competes with Ca^2+^ for the regulatory Ca^2+^-binding site of TnC and negatively influences the actomyosin interaction [11, 21, 64]; therefore, myofibrillar Ca^2+^ sensitivity is *apparently* decreased (increased) when pH is lowered (elevated).

In this short review, we summarize the brief history of the development of measurements of intracellular Ca^2+^ in myocardium using aequorin, and discuss the mechanisms of myocardial contraction and relaxation. In particular, we focus on intrinsically and extrinsically regulated Ca^2+^ transient (CaT) and tension that occur under physiological conditions.

## General scheme of cardiac excitation–contraction coupling

**Fig. 2:**
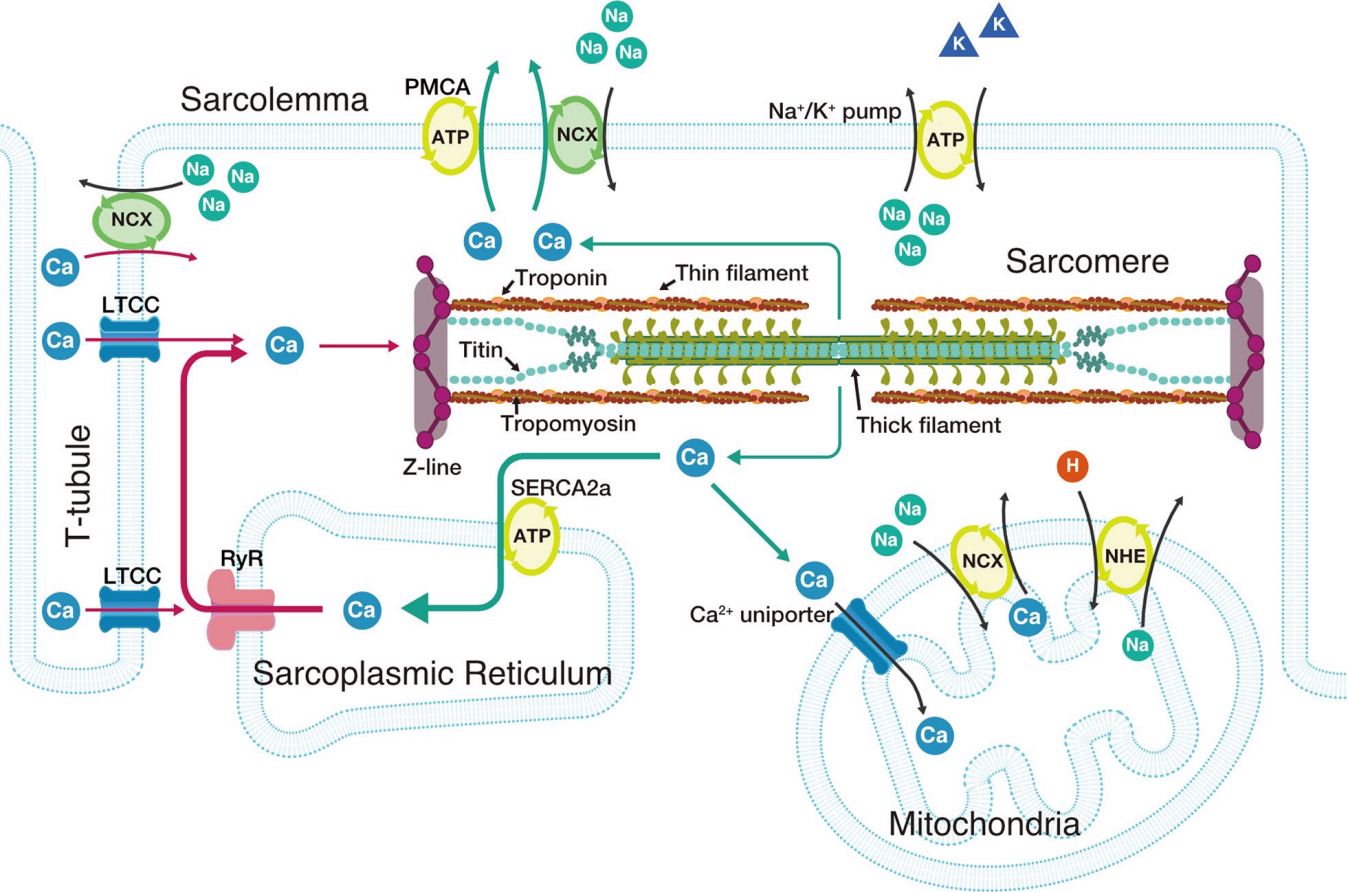
Schematic illustration showing the intracellular structure of a cardiac myocyte. The influx of Ca^2+^ from the interstitial fluid upon excitation causes the release of Ca^2+^ from the sarcoplasmic reticulum (SR). The released Ca^2+^ binds to troponin on thin filaments and triggers sarcomeric contraction (as in Fig. 1). Relaxation occurs as a result of uptake of Ca^2+^ by the SR Ca^2+^ pump, by extrusion of intracellular Ca^2+^ by Na^+^-Ca^2+^ exchangers, and partially by the sarcolemmal Ca^2+^ pump. It still remains elusive whether mitochondria play a significant role in Ca^2+^ handling under physiological conditions. The T-tubules and Z-lines run in parallel, causing Ca^2+^ sparks at/near the Z-lines. Thick and thin filaments, and titin are shown in the sarcomere (for simplicity, two titin molecules per half thick filament are shown). Troponin and tropomyosin exist on thin filaments, regulating actomyosin interaction in a [Ca^2+^]_i_-dependent manner (as in Fig. 1). LTCC, L-type Ca^2+^ channel; RyR, ryanodine receptor; PMCA, plasma membrane Ca^2+^ ATPase; NCX, Na^+^-Ca^2+^ exchanger; NHE, sodium-hydrogen exchanger. Modified from Fig. 2 of [38] in an open access article published by Springer Nature with permission.

First, we briefly summarize the currently accepted mechanisms of cardiac excitation–contraction (EC) coupling (Fig. 2). In mammalian cardiac muscle, contraction is regulated by micromolar concentrations of intracellular Ca^2+^ on a graded basis (e.g., [9, 10, 38, 44]). When the cellular membrane is depolarized coupled with Na^+^ flux, Ca^2+^ enters the myocyte through sarcolemmal L-type Ca^2+^ channels which are localized in the T-tubules. This Ca^2+^ does not directly activate myofilaments, because of its limited content. Instead, it triggers the release of Ca^2+^ from the SR through ryanodine receptors via the Ca^2+^-induced Ca^2+^ release (CICR) mechanism, resulting in the binding of Ca^2+^ to TnC and subsequent binding of myosin molecules to actin (thin filaments), thereby causing thick-thin filament sliding and, accordingly, tension development against various loads (systole). It is important that in cardiac muscle, [Ca^2+^]_i_ is maintained relatively low, i.e., at ~ 0.1 µM at rest, and it is increased to ~ 1 µM via the CICR mechanism prior to the peak of tension development.

[Ca^2+^]_i_ starts to fall prior to myocardial relaxation, and the following four primary mechanisms of Ca^2+^ dynamics are involved in this process: (1) Sequestration into the SR by the Ca^2+^-ATPase pump (i.e., SERCA2a protein), (2) Efflux via the sarcolemmal Na^+^/Ca^2+^ exchanger, (3) Extrusion by the sarcolemmal Ca^2+^-ATPase pump, and (4) Uptake into mitochondria via the Ca^2+^ uniporter. Under physiologic conditions, the mechanisms of (1) and (2) predominantly operate to remove Ca^2+^ from the myoplasm. Once [Ca^2+^]_i_ is lowered to ~ 0.1 µM, Ca^2+^ dissociates from TnC, resulting in detachment of myosin molecules from thin filaments, and therefore, relaxation occurs (diastole).

## Discovery of aequorin: Debut of simultaneous measurements of intracellular Ca^2+^ and tension in muscle preparations

**Fig. 3:**
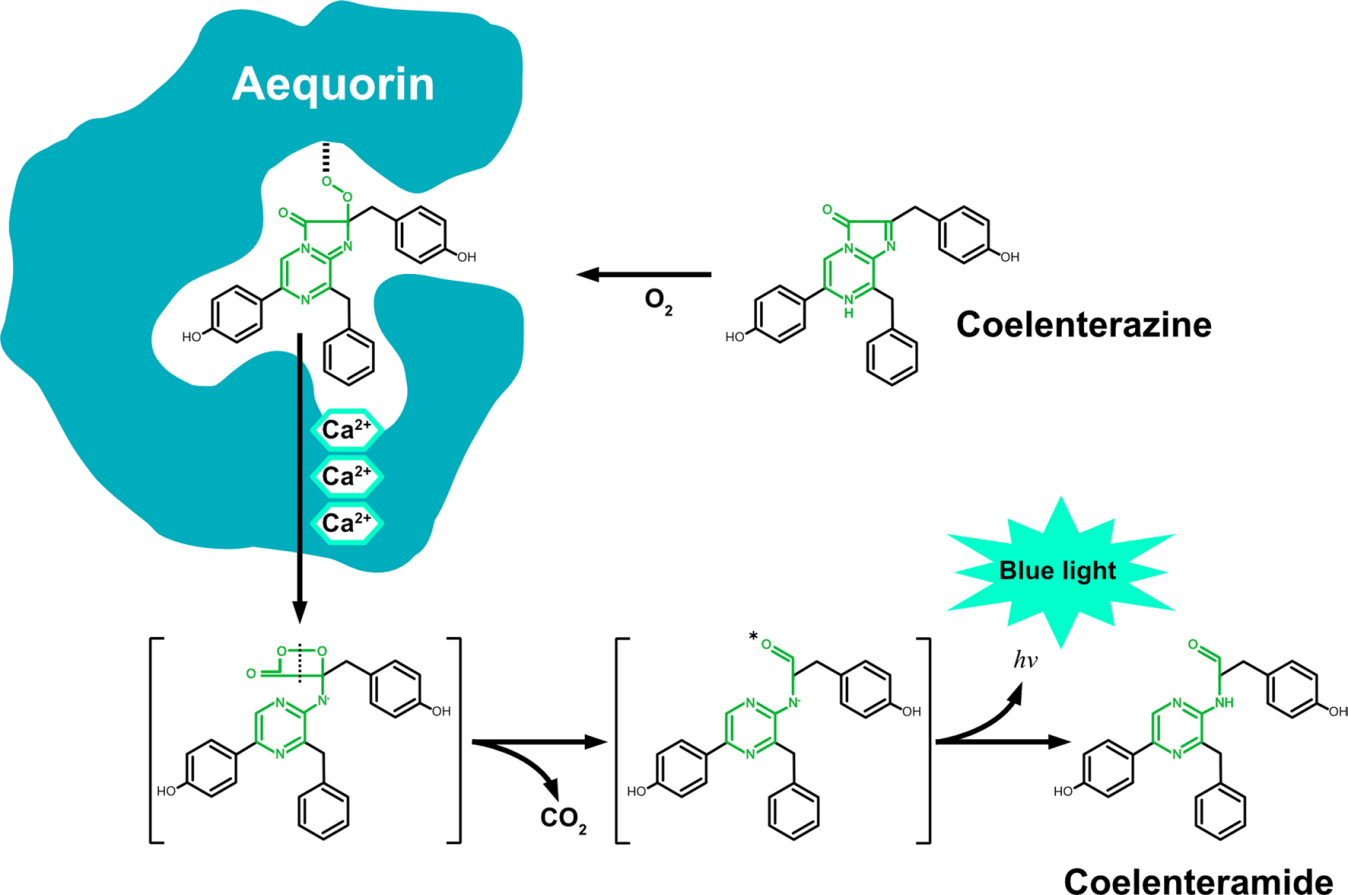
Schematic illustration showing the Ca^2+^-dependent light generation by aequorin. Apo-aequorin is converted to the active form of aequorin when reconstituted by a luminophore coelenterazine in the presence of O_2_. Coelenterazine is oxidized by binding with three Ca^2+^ molecules to the respective EF-hands in aequorin; the subsequent conformational change of the protein in association with the release of CO_2_ produces the singlet-excited coelenteramide that emits blue light (*hv* ~ 470 nm). The portion of coelenterazine where intramolecular changes occur is indicated in green. Modified from Fig. 1 of [70] with permission.

Aequorin is a luminescent protein (molecular weight of 21.4 kDa) which emits blue light (peak wavelength ~ 470 nm) when it binds to Ca^2+^ (Fig. 3). Aequorin consists of apoaequorin (apoprotein), coelenterazine and molecular oxygen. Coelenterazine also has a chemiluminescent reactivity with oxygen. The activation of aequorin by Ca^2+^ catalyzes the oxidation of coelenterazine to coelenteramide, returning to the ground state after emitting blue light (e.g., [5, 12, 61]).

In 1962, Shimomura, Johnson and Saiga isolated and purified aequorin from the jellyfish *Aequorea victoria* (*Aequorea aequorea*) [62]. Their important findings are as follows: (1) Aequorin is sensitive to free Ca^2+^ in the physiologically relevant concentration range, (2) Ca^2+^ is the only cofactor for aequorin required for the emission of blue light, and (3) each aequorin molecule emits light only once. Based on these characteristics, they proposed that aequorin can be a useful tool in measuring intracellular Ca^2+^ of various organisms. In 1968, this proposal was indeed tested by Ashley and Ridgeway who injected aequorin into giant muscle cells (diameter ~ 0.5 − 1 mm) of the barnacle (*Balanus nubilus*), and simultaneously recorded membrane potential, [Ca^2+^]_i_ and tension [7]. They for the first time demonstrated that a dramatic light emission (i.e., CaT) occurred during the early part of active tension production, showing that [Ca^2+^]_i_ needs to be increased to activate contractile proteins. In a later work [8], they more carefully analyzed the relationships between the chemical, electrical and mechanical events of EC coupling, again, in barnacle muscle. Most notably, they reported the following important findings: (1) CaT exhibits a sigmoidal rising phase reaching a maximum soon after the cessation of the stimulus pulse of < 200 ms, and it begins an exponential falling phase during membrane repolarization, (2) CaT is similar in shape to the first derivative of the rising phase of isometric active tension, showing that Ca^2+^ controls the rate of tension development, (3) during the falling phase of tension, aequorin light signals are hardly detected, and (4) there is a linear relationship between the CaT area and peak tension, strengthening the notion that intracellular Ca^2+^ is a primary factor that regulates muscular contractile properties.

In the simultaneous measurement of CaT and tension, a photomultiplier with low dark current is used for detection of aequorin luminescence with low intensity from aequorin-microinjected cells along muscle preparations (see [12] for details). Because of this methodological simplicity, unlike fluorescence Ca^2+^ indicators of which signals decay markedly with time, aequorin enables continuous simultaneous measurement of CaT and tension for up to several hours by applying various mechanical and chemical perturbations. Another distinct advantage of aequorin over other indicators is that the measurement of luminescence is hardly affected by artifacts due to movement of preparations. This property is a great benefit for the measurement of changes in CaT especially when mechanical perturbations, such as length changes, are applied (see below). Moreover, because of its size (21.4 kDa), aequorin stays within the cytosol after microinjection through cellular membranes, hence its luminescence reflects the average cytosolic Ca^2+^ concentration. In contrast, when the acetoxymethyl ester forms of fluorescent Ca^2+^ indicators are used, significant fractions of indicator molecules can be trapped in the intracellular organelles and can bind to myofibrils, making it difficult to accurately quantify the average cytosolic Ca^2+^ concentration (as discussed in [44]).

Shimomura also identified another protein, i.e., the green fluorescent protein (GFP), as a naturally occurring substance in the jellyfish *Aequorea victoria* (*Aequorea aequorea*) (e.g., [12, 61, 62]). Therefore, when GFP is located adjacent to aequorin, aequorin’s blue light (excitation energy) transfers to GFP, resulting in the excitation of GFP. GFP emits green light, and thereafter returns to the ground state. Currently, GFP is widely used in physiology as it allows quantification of protein activity, such as when and where proteins are generated and how various types of proteins or parts of them move within a cell. In 2008, Shimomura received the Nobel Prize in Chemistry with Chalfie and Tsien for the discovery and development of GFP.

## Simultaneous measurements of intracellular Ca^2+^ and tension using aequorin in vertebrate skeletal and cardiac muscles

The Blinks group developed an original method to extract aequorin, independent of the Shimomura group. In their method, aequorin was purified in 10 mM EDTA via a simple but solid five-step procedure [12, 13]. While the studies of Ashley and Ridgway [8] provide valuable insights into the regulation of muscular contraction, it has been pointed out that their results in giant barnacle muscle fibers cannot be directly applied to our understanding of the mechanisms of contraction of vertebrate striated muscle. This is because the ultrastructure of barnacle giant muscle fibers, especially sarcomeres, differs from that of vertebrate striated muscle fibers [33]. In 1978, Blinks, Rüdel and Taylor developed a method to inject aequorin into frog skeletal muscle fibers, having a much thinner diameter (~ 100 μm) compared with barnacle muscle fibers [13]. They simultaneously recorded CaT and tension responses, and revealed physiologically important principles of vertebrate skeletal muscle: viz*.,* (1) CaT appears during the early part of active tension development upon twitch, as in barnacle muscle fibers, (2) CaT and tension start to rise upon tetanic stimulation, with both records remaining at relatively similar levels during tetanus, and (3) exposure of aequorin-microinjected fibers to Ca^2+^-free solution exhibits little or no influence on either CaT or developed tension.

Compared with skeletal muscle fibers, cardiac muscle cells are smaller in diameter (~ 10 − 20 μm) and length (~ 100 μm); therefore, it was considered difficult to microinject aequorin into the cells of myocardial preparations. The first challenge of the simultaneous measurement of CaT and developed tension was performed by Allen and Blinks in frog cardiac muscle [1]. They microinjected aequorin into cells of atrial trabeculae to investigate CaT and tension responses associated with EC coupling. They found that (1) the aequorin light signal increased with increasing either the extracellular Ca^2+^ concentration ([Ca^2+^]_o_) or the stimulation frequency, and (2) application of a β-adrenergic agent (isoprenaline) or a cardiac glycoside (acetylstrophanthidin) increased CaT and tension, but with marked differential effects on the time course of the aequorin light signal (see below for details on the effect of β-adrenergic stimulation on CaT and tension).

These earlier studies which took advantage of the nature of aequorin, greatly advanced our understanding of EC coupling of vertebrate skeletal and cardiac muscles, and hence should be commemorated as a milestone in the history of physiology.

## Launch of research on excitation–contraction coupling in mammalian cardiac muscle

Following publication of the paper by Allen and Blinks in 1978, physiologists’ attention moved towards unraveling the cellular basis of EC coupling of *mammalian* myocardial preparations. Allen and Kurihara started collaborative research on this issue in 1978 at the Department of Physiology of University College London. They improved the microinjection method of aequorin into cardiac cells, originally developed by Blinks, Rüdel and Taylor in 1978 [13], and successfully measured CaT and tension in right ventricular preparations (papillary muscles and trabeculae) from rats and cats [3]. In order to obtain analyzable signal-to-noise ratios of light records, Allen and Kurihara took the following steps: (1) dissolved aequorin (20 − 40 μM) in 75 − 150 mM KCl at pH 7−8 and introduced it into glass micropipettes, and (2) pressure-injected 20 − 60 cells on the upper surface of the preparations and averaged the signals from ~ 30 − 60 contractions. By taking advantage of the sophisticated techniques, they unraveled the following crucially important issues in cardiac physiology: (1) increasing [Ca^2+^]_o_ augments CaT and tension, both in a near linear fashion, (2) the Bowditch effect (also known as the Treppe phenomenon or staircase phenomenon) occurs upon an increase in stimulus frequency (when varied between 1/min and 120/min), (3) adrenaline (between 0.01 and 1 μM) increases both CaT and tension in a concentration-dependent manner, with the effect more pronounced on CaT, and (4) caffeine increases tension in a near concentration-dependent manner, but it lowers the amplitude of CaT, markedly slowing the kinetics of the rising and falling phase of CaT (resulting in prolongation of CaT). In this work, Allen and Kurihara yielded the force-pCa (= − log [Ca^2+^]_i_) curve, and demonstrated that the curve starts to rise at pCa ~ 6.5 and reaches the mid-point at pCa ~ 6.0. It is noteworthy that the curve tended to shift rightward in the presence of adrenaline, suggesting a decrease in myofibrillar Ca^2+^ sensitivity (see below for details).

## Cellular basis of the intrinsic properties of myocardium as revealed by simultaneous measurements of intracellular Ca^2+^ and tension

### I) Frank-Starling effect

**Fig. 4:**
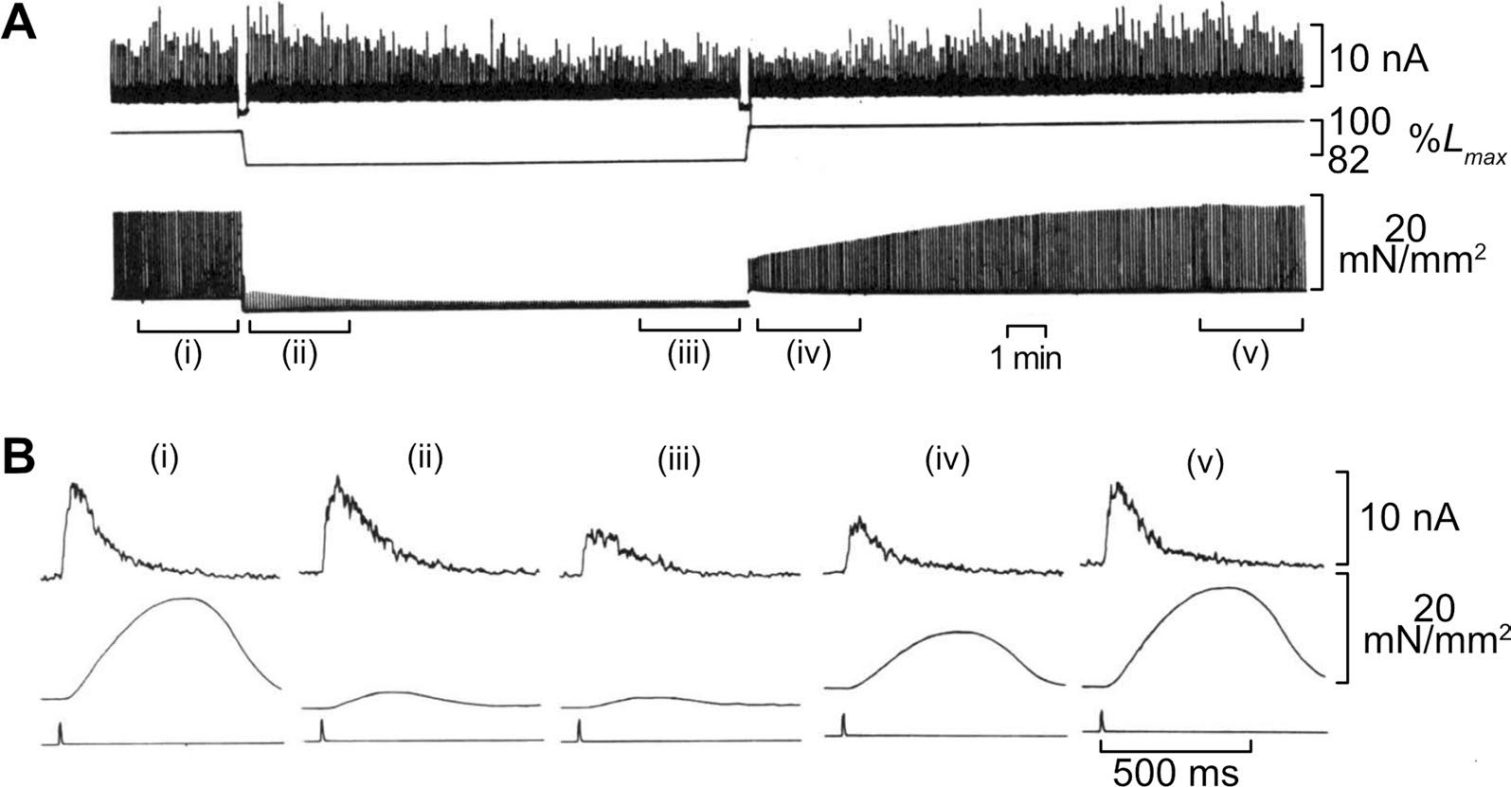
Effects of changes in muscle length on CaT and tension. **A** Experimental records showing the changes in CaT and tension following length changes. CaT and tension were measured at *L*_max_ and when a length change was applied to 82% *L*_max_. Top trace, CaT; middle trace, muscle length; bottom trace, tension. In this experiment, muscle length was changed by a micrometer, and therefore, the CaT recording was discontinued for ~ 20 s during the length change. Cat ventricular papillary muscle was used. **B** Averaged records of CaT (top) and tension (middle) from 32 twitch contractions over the periods shown in **A**, i.e., (i), (ii), (iii), (iv) and (v). The “notch” on each bottom trace indicates the stimulation point. Modified from Fig. 2 of [4] with permission.

At the turn of the twentieth century, Otto Frank in Germany and Ernest Starling in England discovered that an increase in ventricular filling (i.e., elongation of myocardial length) enhances cardiac output, now commonly known as the Frank-Starling Law of the Heart (e.g., [2, 24, 35, 38, 44]). Their “law” describes the heart’s most important intrinsic ability in vivo to immediately alter its contractility, and therefore stroke volume, in response to changes in venous return (hence a fundamental principle in cardiovascular physiology). In 1982, Allen and Kurihara published another milestone paper on the detailed cellular basis of the Frank-Starling mechanism, as investigated by analyzing the changes in CaT and tension during twitch in aequorin-microinjected myocardial preparations, accompanied by changes in muscle length [4]. The physiologically most important and most well-known finding in their 1982 work is that following a sudden change in muscle length, of either shortening or lengthening (by as much as 20%), twitch force is markedly, instantly changed (decreased and increased when shortened and lengthened, respectively) but the peak aequorin light signal barely changes, indicating that the Frank-Starling mechanism is primarily regulated at the myofibrillar level (Fig. 4). Also, Allen and Kurihara carefully analyzed the time-course of both aequorin light signal and tension by varying muscle length, and provided evidence that: (1) the time course of the falling phase of the light signal becomes prolonged upon the shortening of muscle length, and (2) a transient increase in the light signal (designated as “Extra-Ca^2+^” in [45]) appears when muscle length is shortened (hence tension is decreased). To our knowledge, this is the first report in physiology indicating that CaT (i.e., intracellular Ca^2+^ regulation) and tension (i.e., actomyosin interaction) are interrelated in myocardium. As documented by Kentish et al. [37] using skinned rat right ventricular trabeculae, myofibrillar Ca^2+^ sensitivity is increased in response to an increase in sarcomere length by an order of 0.1 μm. Therefore, the findings by Allen and Kurihara [4] indicate a feedback mechanism between Ca^2+^-binding to TnC and muscle length; viz*.*, when intact muscle length is shortened, Ca^2+^ is dissociated from TnC and therefore released in the myoplasm coupled with a decrease in the affinity of TnC for Ca^2+^.

**Fig. 5:**
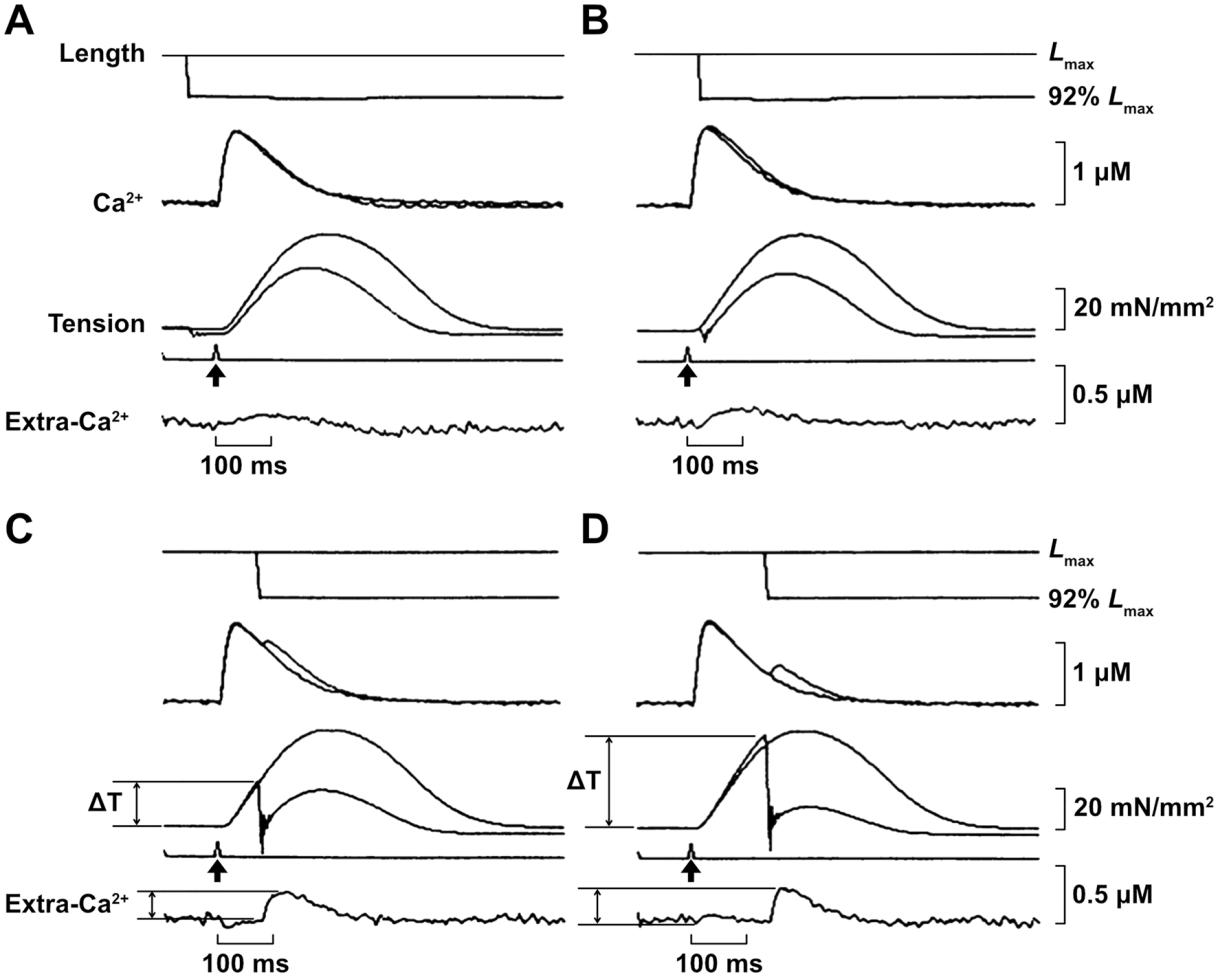
Effects of quick release on CaT and tension during twitch. CaT and tension were measured at *L*_max_ and when a sudden length change was applied to 92% *L*_max_ at various times before (**A**) and after (**B**–**D**) stimulation (indicated by arrows). Ferret ventricular papillary muscle was used. In each panel, two records obtained at *L*_max_ and 92% *L*_max_ are superimposed. Top trace, muscle length; second trace, CaT; third trace, tension; bottom trace, difference of CaT at *L*_max_ and 92% *L*_max_ (i.e., Extra-Ca^2+^ as clearly seen in **C** and **D**). In **A**, muscle length was shortened 50 ms before the stimulus. In **B**–**D**, muscle length was shortened 22, 75 and 138 ms after the stimulus, respectively. Note that Extra-Ca^2+^ becomes greater with greater magnitude of a fall of tension (i.e., Extra-Ca^2+^ greater in **D** than in **C**). ΔT, fall of tension upon shortening. Modified from Fig. 1 of [45] with permission.

The finding of a transient increase in [Ca^2+^]_i_ during CaT in association with quick muscle shortening gained attention from physiologists; however, it was not clarified whether this phenomenon was caused by a decrease in muscle length per se or a resultant fall of tension. To clarify this issue, by taking advantage of the inhibitory effect of 2, 3-butanedione 2-monoxime (BDM) on cross-bridge formation, Kurihara et al. [47] simultaneously measured CaT and tension using aequorin in ferret ventricular muscle. While BDM (10 mM) dramatically suppressed tension development by > 90% (e.g., [31]), it only minimally affected the aequorin light signal. It was found that a sudden change in muscle length (by 8%) caused a transient increase in [Ca^2+^]_i_ (i.e., Extra-Ca^2+^) at various phases during twitch in the absence of BDM; however, although the same magnitude of length change was given, Extra-Ca^2+^ was not observed when BDM was present. It is therefore suggested that a decrease in tension, but not a decrease in muscle length per se, is a cause of Exra-Ca^2+^. Later, Kurihara and Komukai [45] more systematically analyzed the origin of Extra-Ca^2+^ by changing the magnitude of a decrease in tension, but not muscle length, under various conditions in ferret ventricular muscle (Fig. 5). Consistent with the finding of the work by Kurihara et al. [47] using BDM, Extra-Ca^2+^, as well as normalized Extra-Ca^2+^ (i.e., Extra-Ca^2+^/[Ca^2+^]_i_), showed a linear relationship with the magnitude of the decrease in tension. Extra-Ca^2+^ occurred in the presence of caffeine (5 mM), but with a slower time course compared to that observed in the absence of caffeine. Also, stretching the caffeine-treated muscle preparations accelerated the falling phase of CaT, hence a negative deflection of Extra-Ca^2+^ appeared. These findings showed that (1) the origin of Extra-Ca^2+^ is the Ca^2+^ bound to TnC on myofibrillar thin filaments, and (2) the affinity of TnC for Ca^2+^ is under the influence of developed tension, i.e., attachment or detachment of myosin to thin filaments, in physiological twitch contractions in cardiac muscle.

It is noteworthy that Extra-Ca^2+^ is under the influence of intracellular pH. Komukai et al. [42] demonstrated, in aequorin-microinjected ferret ventricular muscle, that acidosis, attained by increasing the CO_2_ concentration in the extracellular solution from 5% (pH 7.35) to 15% (pH 6.89), decreases twitch tension and myofibrillar Ca^2+^ sensitivity, the latter of which is assessed during tetanus in the presence of 5 μM ryanodine (that blocks the release of Ca^2+^ from the SR; e.g. [68, 69, 74]). Here, the expected intracellular pH values are 7.06 and 6.78, for 5% and 15% CO_2_, respectively [57]. It is important that under acidic condition, the magnitude of Extra-Ca^2+^ is decreased, and the slope of Extra-Ca^2+^/[Ca^2+^]_i_ plotted against tension reduction becomes lowered. This finding corroborates the notion that acidosis diminishes Ca^2+^-binding to TnC, as a result of the competition between Ca^2+^ and H^+^ for the regulatory Ca^2+^-binding site of TnC. Accordingly, Extra-Ca^2+^ is decreased, coupled with a decrease in the amount of bound Ca^2+^ to TnC.

### II) Anrep effect

The Anrep effect was first described in 1912 by Gleb von Anrep [72], a Russia-born Egyptian physiologist; this effect constitutes a mechanism by which the heart gradually adapts to an increase in afterload, which occurs after the Frank-Starling effect. It should be addressed that by measuring CaT and tension over a long period of time (~ 30 min), Allen and Kurihara [4] first shed light on the cellular basis of the Anrep effect at the muscle tissue level; they showed that after an increase in muscle length, tension increases dramatically and then shows a slow increase over a period of minutes. It is important that CaT is unchanged immediately after an increase in muscle length; however, it then shows a slow increase with a time course similar to that of the slow tension change. Therefore, while the Frank-Starling effect is regulated at the myofibrillar level, the Anrep effect is likely caused by a gradual change in intracellular Ca^2+^ homeostasis in response to an increase in afterload. Though numerous mechanisms have been reported regarding the rise of CaT (e.g., [14, 36]), it is generally accepted today that a cardinal mechanism is the increased sarcolemmal Ca^2+^ influx through the Na^+^/Ca^2+^ exchanger operating in a reverse mode (e.g., [6, 14]).

## Cellular basis of the neurohormonal regulations of myocardium as revealed by simultaneous measurements of intracellular Ca^2+^ and tension

### β-Adrenoceptor stimulation

**Fig. 6:**
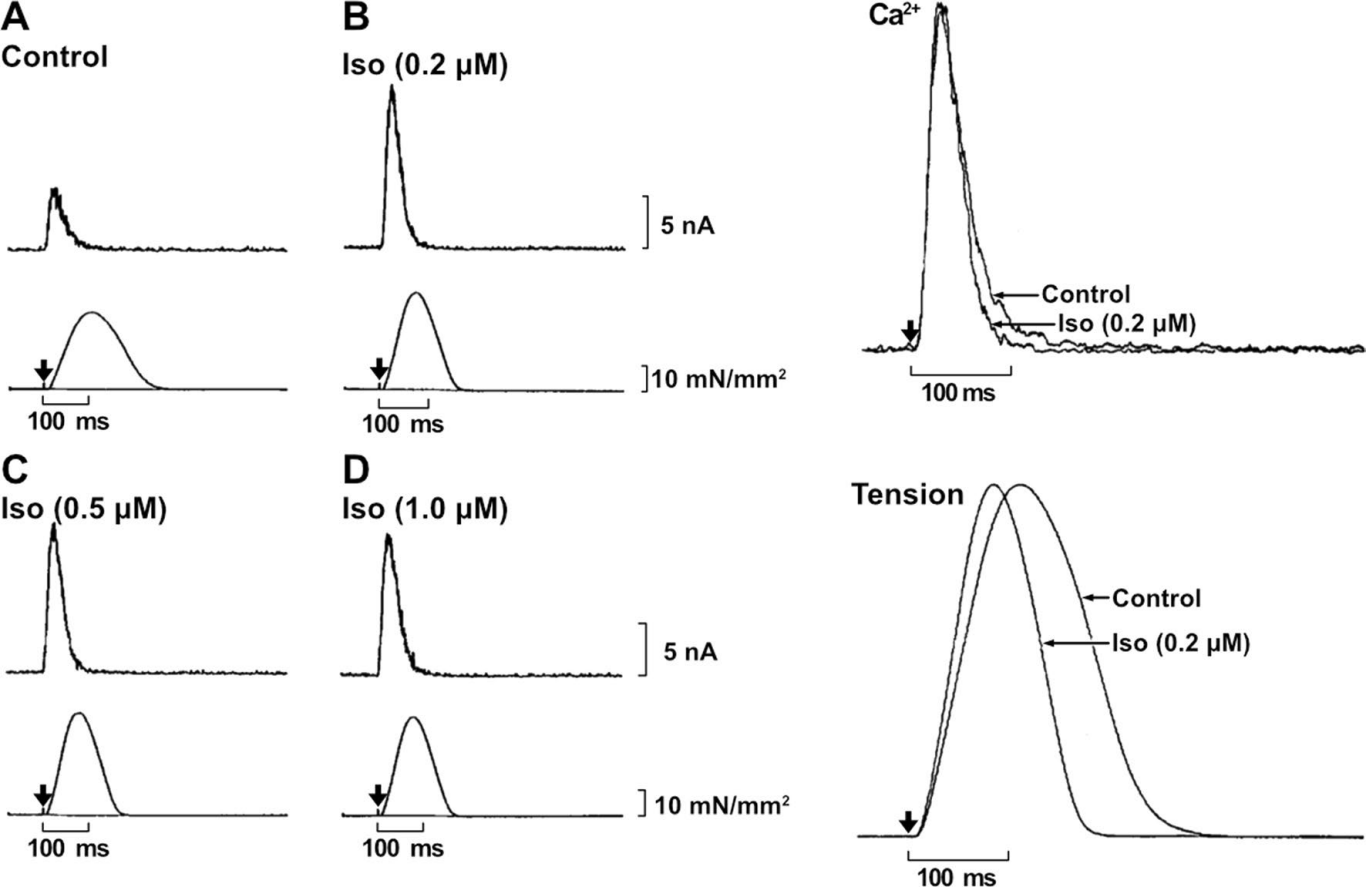
Effects of isoprenaline on CaT and tension during twitch. Left panel (**A**–**D**) shows dose-dependent changes in CaT (top) and tension (bottom). Ferret ventricular papillary muscles were used. Iso, isoprenaline. **A**, control (without Iso); **B**, 0.2 µM Iso; **C**, 0.5 µM Iso; **D** 1.0 µM Iso. Arrows indicate the stimulation point. Right panel indicates the normalized and superimposed CaT (top) and tension (bottom) with and without 0.2 µM Iso. Left and right panels were obtained from the same preparation. Modified from Fig. 6 of [46] with permission.

Mammalian hearts are under the influence of sympathetic and parasympathetic nervous systems, and β-adrenergic stimulation results in marked changes in the contractile behavior of the heart, including increased rates of rise and fall of developed pressure and an increased heart rate. Accumulating evidence shows that β-adrenergic stimulation increases cAMP which activates protein kinase A (PKA) in cardiac muscle, resulting in phosphorylation of many proteins, including L-type Ca^2+^ channels, ryanodine receptors, phospholamban, TnI, myosin-binding protein C and connectin/titin (e.g., [9, 10, 25, 53]). And cAMP is catalyzed by phosphodiesterases to inactive 5’-AMP. Accordingly, myocardial function is associated with increased developed tension and increased rates of rise and fall of developed tension. As first documented by Allen and Kurihara [3] in mammalian cardiac muscle, the changes in intracellular Ca^2+^ handling were thought to be predominantly important causes of these effects. Kurihara and Konishi [46] were the first to carefully investigate the mechanisms of β-adrenergic stimulation-induced changes in aequorin-microinjected rat right ventricular preparations (Fig. 6). Namely, they used adrenaline (0.05–5 µM) and isoproterenol (0.05–1 µM), and found that both agents increased peak twitch tension and accelerated relaxation, especially when [Ca^2+^]_o_ in Tyrode’s solution was low at 0.5 mM, compared with the normal 2 mM. Regarding CaT, both agents increased the peak of the aequorin light signal, and accelerated the falling phase of the light (especially the tail portion of it). These findings led Kurihara and Konishi to conclude that β-adrenergic stimulation increases twitch tension and enhances relaxation predominantly in a Ca^2+^-dependent manner. In order to further investigate the mechanism, they applied the membrane permeable cAMP [dibutyryl-cyclic AMP (DB-cAMP)] or the phosphodiesterase inhibitor 3-isobutyl-1-methylxanthine (IBMX) into Tyrode’s solution, and found that both DB-cAMP and IBMX acted on CaT and tension akin to adrenaline or isoproterenol. To be noted, the findings in the work by Kurihara and Konishi derived nearly a quarter-century ago, provide us with the currently accepted scheme of the intracellular Ca^2+^ regulation upon β-adrenergic stimulation in cardiac cells. Namely, β-adrenergic stimulation increases cAMP, which activates PKA, and as a result, the fall of CaT is accelerated via enhanced Ca^2+^ sequestration into the SR. It is also indicated that the content of Ca^2+^ is increased in the SR during the resting period, and more Ca^2+^ is released from the SR upon subsequent twitch stimulation, making a contribution to an increase in the peak light signal.

In a later study, Okazaki et al. [56] focused on the rising phase of CaT and tension, and found that isoprenaline: (1) increased the peak values of the light signal and tension (as in the 1980 paper by Allen and Kurihara [3]), and (2) reduced time-to-peak light and time-to-peak tension, both of which are now well regarded to be coupled with an increase in the Ca^2+^ influx via L-type Ca^2+^ channels and the subsequent enhancement of the Ca^2+^ release from the SR via the CICR mechanism. Okazaki et al. also confirmed the finding of Kurihara and Konishi [46] that the fall of the light signal and tension is accelerated by β-adrenergic stimulation, indicating rapid and complete cardiac chamber filling during β-adrenergic stimulation in the heart in vivo. Currently, it is well regarded that this enhanced relaxation occurs as a result of enhanced Ca^2+^ sequestration into the SR due to the PKA-dependent phosphorylation of phospholamban (e.g., [43]). Moreover, Okazaki et al. induced tetanic contractions by adding ryanodine (5 µM) in the extracellular solution, and found that the relationship between [Ca^2+^]_i_ and tension shifted to the right, showing a decrease in the sensitivity of contractile proteins to Ca^2+^. To our knowledge, this was the first clear demonstration of the Ca^2+^-desensitizing effect of PKA-dependent phosphorylation of TnI, first reported by Solaro et al. [65], in intact myocardial preparations. It is likely that PKA-dependent phosphorylation of TnI also underlies enhanced relaxation via enhancement of dissociation of Ca^2+^ from TnC, hence detachment of myosin molecules from thin filaments.

Three years after the publication of the well-known paper by Okazaki et al. [56], Hongo et al. [32] discovered that the muscarinic receptor stimulation by application of acetylcholine (ACh) is antagonistic to β-adrenergic stimulation in the Ca^2+^ regulation and tension development in myocardium. Namely, in a well-designed study, Hongo et al. investigated the effects of ACh on CaT and tension in aequorin-microinjected ferret ventricular preparations following application of 0.1 µM isoprenaline. They found that ACh decreased the peaks of both aequorin light signal and tension, and restored the shortened time course of CaT in a concentration-dependent manner (0.01 − 1 µM). They also found that the cross-bridge cycling rate was increased by ~ 20% upon application of 0.1 µM isoprenaline (from 2.73 to 3.25 Hz), which was recovered to the original level by 1 µM ACh. Lindemann and Watanabe [51] reported that ACh reduces the isoproterenol-induced increase in cytosolic cAMP, PKA-dependent phosphorylation of phospholamban and Ca^2+^ uptake into the SR in porcine ventricular preparations. It is therefore likely that ACh reduces the phosphorylation levels of not only phospholamban but also myofilament proteins, such as TnI and myosin-binding protein C, via a decrease in cAMP; this reduction in phosphorylation levels results in the restoration of Iso-induced changes in excitation–contraction coupling and myofilament active properties. The sympathetic and parasympathetic nervous systems may therefore act competitively in the regulation of myocardial function in mammals.

### α-Adrenoceptor stimulation

When the sympathetic nervous system becomes dominant, both α- and β-adrenoceptors are activated throughout the entire body, including the heart. Though the cardiotonic effects of β-adrenergic stimulation were extensively studied in the 1980s and 1990s (see above), information was limited at that time regarding how α-adrenergic stimulation would influence myocardial functions. Endoh and Blinks [22] were the first to shed light on this issue by simultaneously measuring CaT and tension in aequorin-microinjected rabbit papillary muscles. They found that for a given increase in tension, stimulation of α-adrenoceptors (by phenylephrine) produced much less change in the amplitude of CaT than did an increase in [Ca^2+^]_o_, suggesting that myofibrillar Ca^2+^ sensitivity is increased. They also found that stimulation of α-adrenoceptors produced little change or a slight decrease in the duration of CaT and an increase in the duration of tension, while stimulation of β-adrenoceptors (by isoproterenol) significantly decreased the time to peak and duration of both CaT and tension.

Later, Kusakari et al. [48] more carefully investigated the effects of phenylephrine, an α agonist, on intracellular Ca^2+^ regulation (measured by fura-2 fluorescence) and myofibrillar Ca^2+^ sensitivity by using isolated rat ventricular myocytes. Kusakari et al. induced tetanic contractions in the myocytes in the presence of thapsigargin to block the SERCA2a activity, and analyzed myofibrillar Ca^2+^ sensitivity by measuring [Ca^2+^]_i_ and cell length (i.e., Ca^2+^-cell length trajectory) during shortening. They found that while β-adrenergic stimulation with isoproterenol (3 nM) shifted the trajectory rightward, phenylephrine (from 1 to 100 µM) exerted an opposite effect by shifting the trajectory leftward in a concentration-dependent manner, showing an increase in myofibrillar Ca^2+^ sensitivity. Taking advantage of the experimental system allowing for measurements of various intracellular factors, they estimated changes in intracellular pH by analyzing the fluorescence of 2’,7’-bis(2-carboxyethyl)-5(6)-carboxyfluorescein, and found that intracellular pH was increased from ~ 7.1 to ~ 7.2 upon application of 10 µM phenylephrine. Further, provided that ethylisopropylamiloride, an inhibitor of the Na^+^/H^+^ exchanger, or chelerythrine, an inhibitor of protein kinase C (PKC), reversed the Ca^2+^-sensitizing effect of phenylephrine, they concluded that α-adrenoceptor stimulation activates the Na^+^/H^+^ exchanger through a PKC-mediated pathway, resulting in an increase in intracellular pH and, therefore, an *apparent* increase in myofibrillar sensitivity (as noted above).

## Conclusions

The discovery by Ringer of the differential effects between Ca^2+^ and Na^+^ or K^+^ in their action on the isolated frog’s heart led physiologists to consider that Ca^2+^ plays an essential role in cardiac contraction. Eighty years after Ringer’s work, the groundbreaking discovery of aequorin by Shimomura, Johnson and Saiga [62] opened a new era in physiology enabling investigation of critical roles of intracellular Ca^2+^ in amphibian, and then mammalian myocardial preparations under various experimental settings. It must be stressed that as addressed above, experiments using aequorin made great contributions to our understanding of EC coupling in mammalian myocardium, and laid the foundation in the field of research. Also, fluorescent Ca^2+^ indicators, first synthesized by Tsien in 1980 [71], with a binding cavity consisting of four carboxyl groups, and numerous “tetracarboxylate indicators” have been developed, and widely used in myocardial research. Tetracarboxylate indicators can be introduced into cells via incubation with the acetoxymethyl (AM) ester form. Hama et al. [27] perfused the Ca^2+^ indicator Fluo-3-AM into the coronary vasculature via the aorta of the isolated rat heart, and imaged Ca^2+^ waves in the epicardial surface at the cellular level. In a subsequent study, the same group imaged CaT and myocardial membrane potential by using the membrane potential fluorescent agent RH237 under dual-path microscopy [23]. More recently, Shimozawa et al. [63] used another Ca^2+^ indicator, Cal520, for the isolated mouse heart under spinning disk confocal microscopy, and demonstrated that Ca^2+^ waves occur in cardiomyocytes in an independent manner without electric stimulation, indicating that despite the presence of the conduction system, CICR occurs in an independent and autonomous fashion in each cardiomyocyte in the heart. Upon electrical field stimulation, they showed that the individual Ca^2+^ waves are synchronized and, consequently, large CaT appears across the heart, as deduced from the findings of experiments using aequorin by predecessors. Rapid advances in microscopic and molecular biological technologies have now allowed physiologists to perform high-precision analyses of Ca^2+^ dynamics and sarcomeric contractions in cardiomyocytes in the heart of living mice (e.g., [39–41, 63]), creating a new area of research, i.e., in vivo cardiac EC coupling. By fully taking advantage of the emerging technologies, future studies should be directed to further investigating the physiology, pathophysiology and regenerative medicine of the heart, thereby shedding light on the missing links between sub-cellular EC coupling and the cardiac pump function in vivo.

## Data Availability

Not applicable.

## Abbreviations

ACh: Acetylcholine
BDM: 2, 3-Butanedione 2-monoxime
[Ca^2+^]_i_: Intracellular Ca^2+^ concentration
[Ca^2+^]_o_: Extracellular Ca^2+^ concentration
CaT: Ca^2+^ transient
CICR: Ca^2+^-induced Ca^2+^ release
EC coupling: Excitation–contraction coupling
PKA: Protein kinase A
SR: Sarcoplasmic reticulum
Tm: Tropomyosin
Tn: Troponin
