# Correction: Regulation of myocardial contraction as revealed by intracellular Ca^2+^ measurements using aequorin

**DOI:** 10.1186/s12576-024-00915-6

**Published:** 2024-03-26

**Authors:** Satoshi Kurihara, Norio Fukuda

**Affiliations:** Department of Cell Physiology, The Jikei University School of Medicine, 3-25-8 Nishi-Shimbashi, Minato-Ku, Tokyo, 105-8461 Japan

**Correction: The Journal of Physiological Sciences (2024) 74:12 **10.1186/s12576-024-00906-7.

Following publication of the original article [1], the ORCID 0000-0001-8978-8938 for author Norio Fukuda was inadvertently omitted.
